# Abortion associated with postpartum opportunistic bacterial invasion reduces fertility and induces disturbances of reproductive hormones, hematological profile, and oxidant/antioxidant profiles in dairy cows

**DOI:** 10.5455/javar.2023.j721

**Published:** 2023-12-31

**Authors:** Yahia A. Amin, Gamal A. M. Omran, Samer S. Fouad, Mariam A. Fawy, Rawia M. Ibrahim, Fatma Ahmed Khalifa, Rana A. Ali

**Affiliations:** 1 Department of Theriogenology, Faculty of Veterinary Medicine, Aswan University, Aswan, Egypt; 2 Department of Microbiology, Animal Health Research Institute (AHRI), Agriculture Research Center (ARC), Sohag branch, Egypt; 3 PHD of Clinical Pathology of Veterinary Medicine, Qena University Hospital, South Valley University, Qena, Egypt; 4 Department of Zoology, Faculty of Science, South Valley University, Qena, Egypt; 5 Clinical Laboratory Diagnosis, Department of Animal Medicine, Faculty of Veterinary Medicine, South Valley University, Qena, Egypt; 6 Division of Infectious Diseases, Animal Medicine Department, Faculty of Veterinary Medicine, South Valley University, Qena, Egypt

**Keywords:** Abortion, Opportunistic bacterial agents, Fertility, Oxidative profiles, Dairy cows.

## Abstract

**Objective:**

The following study examines for the first time the changes that occur in the post-partum period following abortion in the first trimester of dairy cows using hormonal, hematological, and oxidant/antioxidant profiles. In addition, a bacteriological examination was also performed to explore the role of infections in the complications that occur during this period.

**Materials and Methods:**

One hundred cows were split into two equal groups: The first group enrolled cows that suffered from abortion in the first trimester. The second group enrolled cows that did not experience abortion problems (the control group). Uterine swabs were collected from cows. Blood samples were collected for hormonal, hematological, and oxidative profiles.

**Results:**

Results reveal that *Escherichia coli*, *Staphylococcus* spp*.*, and *Streptococcus* spp*.* are the opportunistic bacteria that were isolated from abortive cows with multidrug-resistant (MDR) characteristics. Red blood cell (RBC) count, hemoglobin, mean corpuscular hemoglobin (MCH), and MCH concentration (MCHC) were significantly higher in the abortive group than in controls in the first 3 days after calving. Conversely, total leukocyte count, platelet count, neutrophils, eosinophils, and immunoglobulin G and M were significantly lower in the abortion group than in controls. The concentrations of estradiol, prostaglandin F2α, oxytocin, and cortisol are significantly increased in the abortive cows, while progesterone is significantly decreased. The levels of malondialdehyde (MDA) were higher in the abortive group, while the levels of superoxide dismutase (SOD), glutathione peroxidase (GPx), and total antioxidant capacity (TAC) were lower.

**Conclusion:**

Abortion during the first trimester of pregnancy increases the risk of postpartum opportunistic bacterial invasion of the uterus. Oxidative stress (OS) and neutropenia are the most important findings that may occur in the postpartum period after abortion and may be due to the abortion itself or its predisposition to opportunistic bacterial invasion of the uterus, which finally causes a fertility reduction.

## Introduction

Bovine abortion causes a considerable economic loss to dairy farms. It is a complex reproductive condition that occurs between 42 and 280 days of pregnancy and is considered one of the most important components of the reduced reproductive performance of high-milking cows [1,2]. It is estimated that each abortion case costs roughly $640.00 in productivity losses [3]. This cost of loss was estimated depending on several factors, such as the time of incidence of abortion during pregnancy, the differences in the performance of predicted cows, the prices of breeding, and replacement decisions. Hanson et al. [4] illustrated that in California herds, the losses were $200 million per year. Recent studies in Algeria indicated that the ratio of abortions in cattle and sheep herds reached 40.29% and 79.41%, respectively [5]. In Mexico, the percentage of cows aborting was 17.7% [6], while in Ethiopia, the overall incidence rate was recorded at 14.30% [7].

Abortions may originate from an infectious agent that can be sporadic or pandemic in nature and is brought on by a variety of pathogens [8–11], such as bacterial, viral, fungal, protozoal, and non-infectious agents [12]. High milk production is one of the non-infectious reasons for abortion [2,13]. In addition, heat stress [1,14], parity [15], previous post-partum disorders [16], and twin pregnancies [2,17] are also non-infectious agents that are responsible for the induction of abortion. Other factors could also induce its incidence or heighten its influence. Age, genetics, or health status represent the most common intrinsic factors, while livestock management, feeding, or stress are the extrinsic ones [9,18].

In cows, some bacterial agents have the ability to cause abortion, such as those that cause diseases such as brucellosis, leptospirosis, listeriosis, and Q fever [19,20]. Sporadic abortions may occur due to infectious organisms such as fungi, *Ureaplasma diversum, Campylobacter fetus*, and *Listeria monocytogenes* [21,22]. While repeated abortion has been reported to be caused by viral diseases including *BHV-1* and *Schmallenberg virus* and coccidian parasites such as *Neospora caninum* in cows [23,24], other bacterial agents, such as *Leptospira spp., Salmonella spp., L. monocytogenes,* and *Campylobacter spp.,* are sporadic causes of bovine abortion and can also transmit harmful zoonotic illnesses [22]. Moreover, many cases of abortion are caused by opportunistic bacterial pathogens, which predominantly remain undetected. These pathogens usually inhibit the host and environment as common inhabitant and sometimes invade the blood stream of the dam till it reaches the placenta, causing sporadic abortion [25].

Increasing understanding of the bacteria responsible for cattle abortion is essential for improving the diagnostic process and identifying new infections [26]. Several factors play a role in the spread of existing pathogens, such as trade globalization, increases in herd size, and environmental change. These factors introduce the disease into regions and animal populations that did not exhibit infection before [27]. The increased microbial load in farms with a large number of heads may help to explain the correlation between abortion and herd size by exposing pregnant females to more bacteria that cause abortions. Additionally, it is more difficult to conduct cleaning and disinfection methods on vast farms, which compromises basic hygiene practices [5]. Other risk variables, such as parity, calving month, pregnancy and lactation stage, mastitis, and prior abortion, have been described for bovine abortion and fetal loss [1,^2^,28].

Oxidative stress (OS) has been linked to pregnancy issues like spontaneous abortion and recurrent pregnancy loss (RPL) [29]. This link originates from the fact that phagocytic cells, which are the primary generators of reactive oxygen species (ROS) and reactive nitrogen species (RNS), are recruited and activated by pro-inflammatory and chemotactic cytokines [30]. The mechanisms of ovarian disruption due to uterine infection and/or biochemical profiles are several and diverse [31]. Nonetheless, the endotoxin lipopolysaccharide (LPS) was strongly evidenced as a key disruptor of ovarian function, as it was determined in the follicular fluid of cases of diseased cattle suffering from uterine infection [32]. Therefore, it was concluded that LPS is directly correlated with bacterial infection and its load [33].

In spite of the fact that many recent studies have been focusing on early embryonic loss, which was recorded with increased incidence [34–36], more research into fetal loss is required because so little is known about the mechanisms that take place in body systems after the incidence of abortion. The risk of abortion is not limited to the loss of the newborn and the loss of a new season of milk yield; it may extend to influencing the post-partum period and reproductive fertility, particularly if associated with infection.

As a result, the current study hypothesized that cows that had abortions are more likely to have infertility in the future due to hormonal insufficiency and OS, especially if the aborted uterus is exposed to invasion by an opportunist bacterial infection after calving. To enhance that understanding, one must comprehend the modifications that take place in the body’s systems following an abortion. Therefore, the aim of the current study did not concern the cause of abortion. Otherwise, the objectives of this study were to investigate the changes that take place in body systems after the incidence of abortion after the first trimester of gestation, its predisposition for opportunist bacterial invasion in the uterus after calving, and its effect on the subsequent fertility of the dairy cows by investigating hormonal, hematological, and oxidative profiles.

## Materials and Methods

The current study’s procedures were carried out in conformity with the guidelines of the Ethics Research Committee of the Faculty of Veterinary Medicine, South Valley University, Egypt (approval No. 74/01.10.2022). The study was performed on a private farm that contains Frisian dairy cows in Qena province, in the south of Egypt. The herd on the farm is composed of 600 cows. The average body condition scores (BCs) of the cows were 3.2 ± 0.12 (scale: 1 = thin, 5 = fat), and they appeared to be in good health (average age, 5–6 years; weight, 350–400 kg) [37]. The herd uses a two-time daily milking schedule (at 6 a.m. and 6 p.m.) with a milking machine. The average milk production per day is 25 ± 0.5 kg of milk, with lactation duration ranging from 270 to 305 days. A total mixed ration (TMR) is used to feed animals, and it is provided three times per day. The TMR used in this study was prepared according to the national research council (NRC) [38]. Mothers and calves were kept separated. Breeding depends on natural mating.

The investigation involved two groups of dairy cows. The first group was composed of 50 cows that had experienced an early abortion (first trimester of gestation). The second group was composed of the same number of cows as the first group, but the cows did not experience abortion problems (control group). Cows were diagnosed as pregnant by transrectal palpation and ultrasonography on days 50 post-serving. Confirmatory pregnancy diagnosis occurs on day 90 post-serving using the same methods of pregnancy diagnosis. Pregnancy losses were considered when the cows that were previously diagnosed as pregnant on day 50 became unpregnant on the pregnancy confirmatory day and were considered to have been clinically aborted. Descriptive epidemiology was the method used in the analysis. Pregnancy loss rates were detected monthly in all animals.

### Sampling for bacteriological isolation

Swabs from the uterus were obtained for a bacteriological examination from all cows (aborted group and control group) in the immediate post-partum period (3 ± 1 day after calving) and every two weeks for two months after parturition. A transcervical-guarded swab from the uterine body of each animal was taken using a previously validated method [39,40]. Briefly, the swab was formed from a long copper wire with a cotton wool tip, wrapped in a metal guard tube with a 58 cm length and an 8 mm external diameter. The swab was autoclaved to sanitize it. To prevent contamination of the swab upon insertion, the guard tube’s distal end was protected by a sterile gelatin half-capsule (Devacaps). Each cow’s vulva was cleaned, and using the rectum as a guide, a swab was pushed via the vagina and cervical canal into the uterine lumen. The swab was introduced into the uterine body through the guard tube, dislodging the gelatin capsule and coming into firm contact with the endometrium 2 cm from the bifurcation of the horns. It was then retracted into the guard and removed from the uterus [33]. The swabs were transported at 4°C in the icebox using a transport medium made of thioglycollate broth and then processed for bacteriological analysis. It was cultured an hour after being collected. Samples were cultured on nutrient broth, nutrient media, Macconkey media, blood agar, Mannitol media, and EMB agar (Lab M Limited, Topley House, 52 Wash Lane, Bury, Lancashire, BL9 6AS, United Kingdom). After a 24-hour period of bacterial culture, the bacteria were identified. Colony characteristics, hemolysis, gram stain, morphology, catalysis, indole synthesis, methyl red, Voges-Proskauer, and citrate production tests were used for identification [40]. For the detection of the resistance of these bacterial agents, in-vitro antibiotic sensitivity testing was performed on all isolates [41]. Isolates with more than three antimicrobial resistances or intermediate susceptibilities were known as multidrug-resistant (MDR) isolates [42]**.** According to clinical and laboratory standards institute (CLSI) (formerly national committee for clinical laboratory standards (NCCLS)) guidelines, the disc diffusion method was used to test antibiotic sensitivity in Mueller-Hinton agar. In order to accomplish this, a separate disc containing Amikacin, Amoxicillin, clavulanic acid, Cefepime, Cefoperazone, Ampicillin/sulbactam, Cefadroxil, Doxycycline, Ciprofloxacin, Rifampicin, Gentamycin, Spiramycin, Nalidixic Acid, and Penicillin was employed.

### Breeding soundness examination

All cows (aborted cows and control ones) were subjected to breeding soundness examination after delivery with the administration of PGF2α injection (2 ml of estrumate, 500 mcg cloprostenol, per cow through intramuscular injection). Duration to 1st estrus (d); days open (DO) (d) (the end of the voluntary waiting period, which was considered 50 days to successful insemination); and the number of services per conception were the breeding parameters that were investigated.

### Sampling for hormonal, hematological, and OS testing

Blood samples (20 ml) were collected from the animals’ jugular veins [43] from all cows one day after parturition, three days after parturition, and 15 days after parturition. Blood was collected into two different types of vacutainer collecting tubes: one type was coated with ethylene diamine tetraacetic acid (EDTA) as an anticoagulant for evaluating the hematological parameters, and the other type was plain vacutainer tubes for separating serum to measure hormonal and oxidant/antioxidant parameters. Separate serum samples were kept at -20°C pending further examination.

### Hematological analysis

Using an automated hematology analyzer (Scil Vet ABC Hematology Analyzer, Scil Animal Care Company, USA), the following parameters for the hematological analysis were determined: Red blood cell count (RBC), packed cell volume (PCV), hemoglobin concentration (Hb), platelet count, mean corpuscular volume (MCV), mean corpuscular hemoglobin (MCH), MCH concentration (MCHC), and white blood cell count (WBC). Blood smears stained with Giemsa stain were used to manually calculate the differential leukocyte count, including the percentages of neutrophils, lymphocytes, monocytes, basophils, and eosinophils. The serum immunoglobulin (Ig) G and M levels were measured as previously described [44].

### Hormonal detection

Progesterone (P4) and estradiol (E2) concentrations were determined using an ELISA Kit (Diaplus, North York, Ontario, Canada) as described by Heidari et al. [45]. Oxytocin (OT) was examined using the OT ELISA Kit (Catalog No. E-EL-0029) product of Elabscience® (USA). Prostaglandin F2 alpha (PGF2α) was analyzed using the Human PGF2α (Prostaglandin) ELISA Kit (Catalog No: E-EL-H1841) in accordance with the manufacturer’s instructions (Elabscience^®^, USA). Cortisol was detected by commercially available enzyme-linked immunosorbent reagent kits (Diagnostic Biochem Canada, Canada).

### Oxidant and antioxidant detection

The Spectro Ultraviolet spectrophotometer (Labomed, Inc., Los Angeles, CA, USA) was used to determine the serum concentration of malondialdehyde (MDA) colorimetrically as an indicator of lipid peroxidation (Biodiagnostic commercial assay kits, Cairo, Egypt). The same device was used for the detection of the plasma levels of superoxide dismutase (SOD), glutathione peroxidase (GPx), and total antioxidant capacity (TAC) as antioxidant biomarkers (biodiagnostic commercial assay kits).

### Statistical analysis

The data are described as means ± SE. Data analysis was performed using SPSS Statistics 19 software (IBM, USA) using the analysis of variance technique (ANOVA). A general linear model ANOVA with repeated measures was used to evaluate significant differences. As the variation of time and its effect on the results were not significant (*p* > 0.10), time trends were excluded from the final model. Dependent variables were concentrations of reproductive hormones, hematological parameters, and oxidant and antioxidant levels between the abortion and control cows. Differences were considered significant at *p* ≤ 0.05.

## Results

### Bacterial isolation and sensitivity test

**Figure 1.**
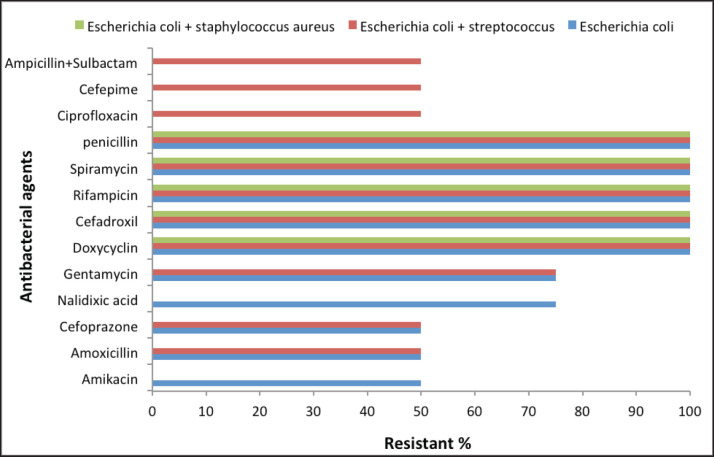
Antibacterial sensitivity test of *E. coli* and mixed bacterial isolates recovered from uterine discharge samples against different antibacterial agents.

Bacteriological examination indicates that abortive cows suffer from different types of bacterial infections (*Escherichia coli*, *Staphylococcus spp.*, and *Streptococcus spp.*) compared to control cows that exhibit no bacterial growth. The isolated bacteria are either single, such as *E. coli* (24%, *n =* 12 cows), or mixed with each other, such as *E. coli* and *Staphylococcus spp.* (50%, *n =* 25 cows), or *E. coli* and *Streptococcus spp.* (26%, *n =* 13 cows). The antibiotic sensitivity test indicates that all types of isolated bacteria are MDR bacteria (Fig. 1).

### The adverse effect of abortion on the fertility profile

The duration of the first estrus, the DO, and the number of services per conception exhibit a significant increase in the group of cows having abortions compared to the control group (Table 1).

### Hematological analysis

The hematological parameters of cows suffering from abortion compared to control cows are presented in Table 2. Abortive cows demonstrated a statistically significant (*p* < 0.05) drop in RBC, hemoglobin, MCH, and MCHC on day 15 postpartum compared to the other healthy cows; in addition, the PCV was decreased, but the decrease was not significant. The values of mean cell volume (MCV) increased on the 15th postpartum day of abortive cows, but the increase was not significant. The values of the total leukocytic cell count, the platelet count, and the percentages of neutrophils and eosinophils on day 15 postpartum in the abortive cows were significantly decreased compared to their values in control cows. In contrast, lymphocytes in the abortive cows were significantly increased compared to the control cows, while monocytes and basophils showed no difference between the two groups. Checking of IgM and IgG reveals that there is a significant decrease in their values in abortive cows compared to control ones.

### Hormonal detection

Examination of the hormonal profile indicates that the concentrations of E2, PGF2α, OT, and cortisol are significantly increased in the aborting cows compared to the control group. In contrast, P4 is significantly decreased in the aborting cows compared to the control group (Table 3).

### Oxidant and antioxidant detection

The levels of MDA in the abortion group have significantly increased as compared to the healthy group at all the time points in the study. Biomarkers of the antioxidant profile, including SOD, GPX, and TAC levels, in the abortive group were significantly lower than those in the control animals at all time points of the study (Table 4).

**Table 1.** Adverse effects of abortion on the fertility profile of dairy cows.

Item	Abortive cows (*n =* 50)	Control cows (*n =* 50)
Duration to 1st estrus	95.36 ± 1.16*	36.29 ± 1.06
Days open	125.31 ± 2.11*	66.15 ± 1.32
Number of services per conception (n)	2.15 ± 0.26*	1.20 ± 0.18

Values are expressed as Mean ± SE. Asterisks (*) indicate statistically significant (*p* < 0.05)

## Discussion

Cow abortions continue to be a serious problem for dairy farms, where there could be large financial losses. The current study is concerned with the detection of abortion in the first trimester of gestation. According to a previous study conducted in Mexico, cow pregnancies were lost the most often between days 45 and 90 of gestation [46]. Some reports showed that most abortion cases occur in the second trimester of pregnancy [47], while others showed that they occur mostly in the third trimester [48,49], with the second trimester reported to be of the highest risk [4,50]. These differences in the timing of abortion might be due to the difficult diagnosis of abortion during the first trimester of gestation, and some causes of abortion in cattle were gestation stage-specific [51].

The diagnosis of bovine abortion is challenging, and despite thorough laboratory testing, the variety of causes cannot be recognized [9,52]. The intricacy of the etiology of bovine abortion has led to its classification as a syndrome [53]. The frequent autolysis of the fetus and placenta prevents the successful detection of infectious agents by preventing the observation of lesions. Another challenge in diagnosing bovine abortion, particularly in epidemic outbreaks, may come from the transmission of many abortigenic agents within a single herd [54]. In addition, nonpathogenic or opportunistic bacteria are frequently present in bacterial cultures from fetal tissues and placentas [9], which makes it challenging to interpret the results, particularly in situations where there are no lesions indicative of bacterial infection.

In the current study, the opportunistic bacteria isolated from the abortive cases of cows were *E. coli*, *Streptococcus spp.*, and *Staphylococcus spp.* These findings are in line with those shown by Macas-Rioseco et al. [47], who reported that among cases that had an etiologic diagnosis, 94.4% were caused by infectious agents, and 21.5% of these cases were associated with opportunistic bacteria. The authors also indicated that the most common opportunistic pathogens were *Escherichia coli*,* Streptococcus spp.*,* and Staphylococcus spp*.

**Table 2.** The hematological parameters of cows with history of abortion compared to control cows.

Items	Abortive cows (*n =* 50)		Control cows (*n =* 50)	
	1–3 days after calving	15 days postpartum	1–3 days after calving	15 days postpartum
RBCs (×10^6^/μl)	6.5 ± 0.12 ^a^	5.5 ± 0.11 ^b^	5.7 ± 0.14 ^c^	5.72 ± 0.18 ^c^
Hemoglobin (g/dl)	16 ± 0.11 ^a^	11 ± 0.12 ^b^	13 ± 0.13 ^c^	13.15 ± 0.11 ^c^
PCV %	44 ± 0.12 ^a^	34 ± 0.17 ^b^	35 ± 0.18 ^b^	35.6 ± 0.17 ^b^
MCV (fl)	68 ± 0.13 ^a^	62 ± 0.14 ^b^	61 ± 0.11 ^b^	61.5 ± 0.12 ^b^
MCH (pg)	25 ± 0.18 ^a^	20 ± 0.19 ^b^	23 ± 0.14 ^a^	24.02 ± 0.12 ^a^
MCHC (gl)	364 ± 0.11 ^a^	324 ± 0.14 ^b^	371 ± 0.17 ^a^	372 ± 0.17 ^a^
Total leukocyte count (×10^3^/μl)	10.60 ± 0.12 ^a^	6.85 ± 0.11 ^b^	11 ± 0.15 ^a^	11.22 ± 0.13 ^a^
Neutrophils %	26 ± 0.17 ^a^	20 ± 0.19 ^b^	52 ± 0.18 ^c^	51 ± 0.17 ^c^
Eosinophil %	02 ± 0.11 ^a^	02 ± 0.13 ^a^	06 ± 0.14 ^b^	06 ± 0.14 ^b^
Basophil %	00	00	00	00
Lymphocytes %	70 ± 0.16 ^a^	78 ± 0.19 ^a^	42 ± 0.17 ^b^	43.02 ± 0.15 ^b^
Monocyte %	02 ± 0.11	00	00	00
Platelets count (×10^3^/μl)	210 ± 0.16 ^a^	170 ± 0.14 ^b^	220 ± 0.18 ^a^	221 ± 0.17^ a^
IgM (mg/dl)	6.5 ± 0.13^ a^	3.0 ± 0.11 ^b^	5.0 ± 0.12^ c^	5.12 ± 0.10 ^c^
IgG (mg/dl)	16 ± 0.15 ^a^	13 ± 0.16 ^b^	30 ± 0.14 ^c^	32.01 ± 0.16 ^c^

Values are expressed as Mean ± SE. Means bearing different superscripts in the same row differ significantly (*p* < 0.05).

**Table 3.** The hormonal profile of cows with history of abortion compared to control cows.

Items	Abortive group (*n =* 50)		Control group (*n =* 50)	
	1–3 days after calving	15 days postpartum	1–3 days after calving	15 days postpartum
Progesterone (ng/ml)	0.8 ± 0.15 ^a^	0.17 ± 0.14 ^b^	0.95 ± 0.16 ^a^	2.66 ± 0.16 ^c^
Estradiol (pg/ml)	16.15 ± 0.15 ^a^	14.15 ± 0.15 ^a^	9.28 ± 0.18 ^b^	5.28 ± 0.18 ^b^
Oxytocin (pg/ml)	10.74 ± 0.11^a^	8.74 ± 0.11 ^a^	4.55 ± 0.12 ^b^	2.57 ± 0.13 ^b^
Prostaglandin F2 α (ng/ml)	7.4 ± 0.13 ^a^	6.4 ± 0.12 ^a^	1.3 ± 0.14 ^b^	0.3 ± 0.14 ^b^
Cortisol (ng/ml)	2.51 ± 0.14 ^a^	2.14 ± 0.14^ a^	1.50 ± 0.16 ^b^	0.89 ± 0.16 ^b^

Values are expressed as Mean ± SE. Means bearing different superscripts in the same row differ significantly (*P* < 0.05).

**Table 4.** Mean activity of the oxidant/antioxidant profile of cows with history of abortion compared to control cows.

Items	Abortive group (*n =* 50)		Control group (*n =* 50)	
	1–3 days after calving	15 days postpartum	1–3 days after calving	15 days postpartum
Malondialdehyde (MDA) (nmol)	4.5 ± 0.02 ^a^	4.0 ± 0.02 ^a^	3.5 ± 0.05 ^b^	3.4 ± 0.05 ^b^
Superoxide dismutase (SOD) (U/ml)	2.2 ± 0.03 ^a^	2.1 ± 0.03 ^a^	3.2 ± 0.01 ^b^	3.1 ± 0.01 ^b^
Glutathione peroxidase (GPX) (U/ml)	815 ± 0.06 ^a^	820 ± 0.06 ^a^	1005 ± 0.02 ^b^	1000 ± 0.02 ^b^
Total antioxidant capacity (TAC)(mmol/ml)	0.9 ± 0.12 ^a^	1.0 ± 0.12 ^a^	1.5 ± 0.11 ^b^	1.7 ± 0.12 ^b^

Values are expressed as Mean ± SE. Means bearing different superscripts in the same row differ significantly (*P* < 0.05).

As sporadic abortifacients in cattle, bacteria like *E. coli*, *Streptococcus spp.*,* Staphylococcus spp.*, and *Mannheimia spp.* have also been identified in prior reports and linked to suppurative lesions in the placenta, lungs, and occasionally other fetal tissues [9,53].

It’s interesting to note that new research has questioned the extent to which *E. coli* is linked to uterine disease. A recent study indicated that *E. coli* was found to be responsible for causing postpartum uterine disease such as postpartum endometritis in dairy cattle, and sometimes its presence was associated with other types of bacterial agents such as *Staphylococcus spp.*, *Streptococcus spp.,* and *Klebsiella* spp*.* [55]. However, some studies have found a tenuous link between uterine illness or infertility and the presence of *E. coli* at 35 days in milk (DIM) [56,57]. It is essential to distinguish between the presence of *E. coli* in the uterus at 35 DIM and the importance of *E. coli* in the uterus shortly after parturition, where accompanying uterine disease and infertility exist [58,59]. Furthermore, in the current study, it is not confirmed whether the opportunistic bacteria isolated from the abortive cases were the cause of abortion in the first trimester of gestation or whether the opportunistic bacteria invaded the uterus during or after abortion.

Losing a pregnancy has a significant detrimental influence on reproductive effectiveness and, consequently, on-farm profitability [60]. According to Wijma et al. [61], cows that have abortions have a higher risk of having another one if they conceive again, a reduced risk of pregnancy by 400 DIM, and a greater risk of culling from the herd than non-aborted cows. Abortions can also result in additional health issues such as retained placenta, metritis, endometritis, and pyometra, which can have an adverse effect on fertility, milk production, and productive life [62,63]. In the current study, compared to the control group, aborted cows had a significantly longer time to the first estrus, more open days, and more services needed for each conception. These results are consistent with those of earlier research that illustrated that the inflammatory condition causes an increase in days to first service, decreased pregnancy/AI, and increased DO [33,64]. In the study performed by Albuja et al. [46], the effects of abortion indices on three reproductive parameters, including calving interval (CI), DO, and dry days (DD), were determined. The findings of the later study revealed a positive correlation between the frequency of simulated abortions and the lengthening of the average days for all three parameters.

The increased duration of the first estrus after abortion may be related to ovarian inactivity and/or anovulation. Retardation of ovulation and sometimes anovulation in cows result from uterine infections resulting from several causes, such as slower growth of the postpartum dominant follicle in the ovary, lower peripheral plasma estradiol concentrations, and perturbation of hypothalamic and pituitary function [65]. Ovulatory postpartum follicles are characterized by reduced synthesis of androstenedione and estradiol [66,67]. Prolonged postpartum anovulation, which is considered the main cause of infertility, usually results from the combination of the incidence of uterine disease and the presence of smaller, slower-growing follicles on the ovary, which exhibit reduced steroidogenesis [68]. The mechanisms of ovarian disruption due to uterine infection and/or biochemical profiles are several and diverse [31]. Nonetheless, the endotoxin LPS was strongly evidenced as a key disruptor of ovarian function, as it was determined in the follicular fluid of cases of diseased cattle suffering from uterine infection [32]. Therefore, it was concluded that LPS is directly correlated with bacterial infection and its load [33,69].

Till now, there have been no studies concerned with the variations that occur in the postpartum period of abortive cases of dairy cattle in the first trimester of gestation. The current study is the first to evaluate the hematological differences in abortive cases of cows in the first trimester of gestation that are associated with opportunistic bacterial infections. Due to this shortage of data about this topic, the authors of the current study compared the current findings with those mentioned in the previous studies concerned with abortion due to Leptospira, which can cause abortion at any stage of gestation.

RBC, Hb, and MCV values in the current study’s abortive cows were much lower than those in control cows, which was statistically significant. These outcomes resemble those that have been noted in cows with Leptospira seropositivity [70] and in goats [71]. Moreover, similar results were found by Ata et al. [72], who showed that the RBC counts and hemoglobin were significantly lower in two groups of women that suffer from early pregnancy loss and a threatened abortion, respectively, compared to the healthy control group.

In the current findings, the values of MCH, MCHC, total leukocytic count, platelet count, neutrophil count, and eosinophil count were significantly decreased compared to their values in control cows. In contrast, the percentages of lymphocytes were significantly higher compared to their values in the controls. These results are almost identical to those found in the cases of cows having an abortion due to leptospira infection [70].

The association of abortion in the first trimester with the invasion of opportunistic bacteria indicates that these animals suffer from immunosuppression. This point of view is confirmed by Dirandeh et al. [73], who reported that cows that were pregnant and lost their embryos had reduced ISG15 expression (Type I interferons play a role in defense mechanisms to protect the host), the expression of inflammatory genes, such as interleukin-1, is elevated, and eicosanoids (polyunsaturated fatty acids play important roles in endocrine systems) associated with inflammatory prostaglandin responses are expressed differently. The key innate immune cells that help the body recover from bacterial infections are neutrophils [74]. The decrease of neutrophils in abortive cows is particularly severe, claiming that neutropenia is compatible with a true pathogenic illness. The low percentage of neutrophils in the current study may explain the invasion of opportunistic bacteria in abortive cows. Therefore, among the significant discoveries found in this investigation is the association between abortion and neutropenia. Severe neutropenia is either primarily due to reduced myelopoiesis or secondary to severe inflammation related to consumption due to increased peripheral demand [75,76]. This latter state may be caused by a serious systemic inflammatory reaction, typically of bacterial origin (such as septicemia), and the recruitment of neutrophils in organs with focal inflammatory alterations, which results in macroscopic evidence of the neutrophils aggregating in inflamed locations (e.g., purulent inflammation, abscesses, etc.). Therefore, the isolation of the opportunistic bacteria from abortive cows is favorable to the presence of a moderate or severe systemic inflammatory response.

It is acknowledged that lymphocytes continue to predominate in mature cattle, with a neutrophil-to-lymphocyte ratio of roughly 1:2. This ratio (also known as the stress leukogram) might change for a number of reasons. Infections caused by bacteria, viruses, protozoa, parasites, and fungi have all been linked to inflammatory granulocytosis [77]. Dairy cows with increased lymphocytes and reduced neutrophils after an abortion due to opportunistic bacteria in a ratio exceeding 1:2 (1:3.9) may explain the invasion of opportunistic bacteria to cause abortion and increase the risk of animal immunosuppression before parturition and during the early postpartum.

In the current findings, the values of platelet counts were significantly decreased. These results could be explained by an increase in platelet consumption for their destruction during the chronic inflammatory process brought on by the bacterial infection [78]. However, as none of the earlier studies looked into these variables, it is impossible to make meaningful inferences.

A precise immunologic exchange at the endometrial-maternal-fetal immunological interface is necessary for a successful pregnancy. These maternal-fetal immune interfaces are extremely elaborate during early gestation and comprise a great deal of immunocytes, involving innate lymphocytes (ILC), macrophages, decidual dendritic cells (DCs), and T cells. A balance between the inflammatory response and immunological tolerance is established in large part by these cells [79]. Checking of IgM and IgG in the current study reveals that there is a significant decrease in their values in abortive cows at day 15 postpartum compared to control ones. Previous research suggests that diseases affecting the endometrial immunological microenvironment are linked to serious, life-threatening reproductive abnormalities, including recurrent spontaneous abortion (RSA) and implantation failure (RIF) with an unknown cause [80].

The contraction of the cow’s myometrium is directly and indirectly influenced by P4, E2, OT, and PG [81]. Ovarian E2 and P4 both influence the pituitary’s secretion of OT [82]. The regulation of OT receptors in the myometrium can trigger strong utero-muscular contractions by increasing estrogen levels and triggering PGF2α release [83,84]. Prostaglandins cause myometrial contractions associated with the lysis of the corpus luteum, which secretes relaxin and reduces P4 concentrations [84]. Any hormone imbalance in cows can lead to cow/calf losses and reduced reproductive health, which can have negative direct and indirect consequences on productivity. In the current study, high levels of PGF2, E2, cortisol, and OT are likely to rise in aborting cows at all-time points while P4 falls.

PGF2α is an uterotonic mediator responsible for increasing contraction of the uterus; therefore, it is used for induction of labor and estrus synchronization and has a positive feedback mechanism with the production of luteinizing hormone [18]. The prevalence of cow abortions has been linked to the increase in prostaglandin [85]. The mean prostaglandin concentrations in the current findings in abortion cases are significantly higher than those of healthy cows. These results are almost similar to those mentioned in the study that investigated protozoal invasion of the placenta and heart. This study found that the prostaglandins generated due to the invasion of the placenta and heart by protozoa can result in cow abortion [86]. Other investigations have demonstrated the detrimental consequences of high PGF2α concentrations on the survival of the embryo, including embryo deaths, malformations, and stillbirths [87,88]. Additionally, luteolysis brought on by prostaglandin-induced alterations in luteal circulation is typically followed by decreased P4 synthesis, which results in abortion [88].

The P4 hormone is in charge of preparing the uterus for pregnancy and sustaining it [89]. Consequently, there is a considerable change in P4 content in cows during abortion [85]. Low P4 levels have been associated with fetal deaths because of their links to inadequate nutrition, which leads to a negative energy balance. This interferes with the microenvironment in the uterus and results in abortions in cows [88]. In addition, stress causes increased P4, which stimulates immunosuppressive protein accumulation in the uterine lumen, leading to susceptibility to persistent bacterial infections [84,90]. Moreover, Sina et al. [64] found that P4 concentration was lower in the first and second estrous cycles after calving in inflamed cows, which also have smaller corpora lutea (CL) compared to healthy cows. Therefore, the authors concluded that prolonged days to the first service, decreased pregnancy/AI, and increased DO usually occur in cows suffering from inflammatory conditions [33,64].

Numerous investigations revealed that excessive OS from the placenta and/or maternal tissues was linked to pregnancy problems [91]. Increased ROS and antioxidant system depletion lead to the development of an oxidative situation [92]. Embryogenesis defects, unexplained RPL, and spontaneous abortion are all caused by the development of aberrant OS [93,94]. In reaction to OS, pregnancy issues like spontaneous abortion and RPL might also appear [95].

**Figure 2.**
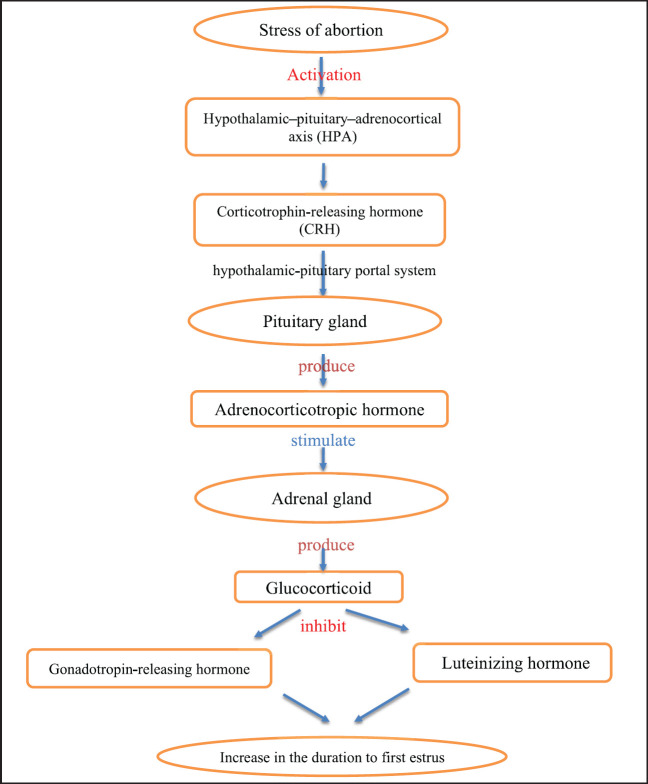
The role of stress of abortion in increase the duration to first estrus.

The hypothalamic-pituitary adrenocortical axis (HPA), which regulates hormone production, is stimulated during stress reactions [96]. The hypothalamus secretes corticotropin-releasing hormone (CRH). This CRH is transmitted through the hypothalamic-pituitary portal system and stimulates the pituitary gland to produce the adrenocorticotropic hormone (ACTH). ACTH stimulates the production of glucocorticoids by the adrenal gland [97]. Glucocorticoids are characterized by their indirect inhibitory effect on gonadotropin-releasing hormone (GnRH) and luteinizing hormone (LH). These effects are mediated by the KNDy cells (Kisspeptin, neurokinin B, and dynorphin neurons), which possess glucocorticoid receptors and transmit the glucocorticoid signal to the GnRH neurons in the hypothalamus [97–99]. This may give an explanation for the causes of the significant increase in the duration of the first estrus and the increased DO in the group of abortive cows in the present study compared to the control group (Fig. 2). This point of view is evidenced in the present study, in which the serum cortisol concentrations were found to increase in abortive cows compared to controls. These findings are supported by Paiano et al. [100], who illustrated that several physiological alterations occur in the transition period of dairy cows, such as a decrease in body weight and an increase in serum cortisol concentrations.

In the current study, a considerable rise in the levels of MDA was observed at all-time points in abortive cows compared to healthy ones. Moreover, different antioxidant components such as SOD, GPX, and TAC exhibited a marked reduction in their levels in the abortive group compared to control animals. These results are consistent with the earlier study, which showed that lactating dairy cows that retained pregnancy had clearly higher levels of GPX, SOD, and TAC activity than those that lost their embryos between days 16 and 32 [73]. Furthermore, cows that suffered from embryonic loss after timed artificial insemination (TAI) had higher MDA concentrations compared to cows that maintained their pregnancy. Nazari et al. [101] stated that cows that lost their pregnancy had lower activities of the postpartum GPX, SOD, and TAC compared to those who maintained pregnancy at days 32 and 60 after artificial insemination (AI). Furthermore, normal luteal activity, earlier resumption of cyclicity, decreased pregnancy loss, and increased conception rate were observed to occur in Holstein dairy cows, which exhibited greater antioxidant levels in the early postpartum period.

GPX and SOD are the two prominent members of the enzymatic antioxidant system responsible for the protection of the cells against ROS. SOD is one of the primary antioxidant enzymes, and it is thought that SOD activity is the first line of defense against OS. SOD comes in three different isoforms, including SOD1, SOD2, and SOD3. A study conducted by Ghneim et al. [102] found a decrease in SOD1 and SOD2 activity in women with RPL compared to healthy control women. This is almost in agreement with the findings of the current study.

## Conclusion

The incidence of pregnancy loss may be the key reproductive factor influencing how profitable intensive dairy farming systems are. New insights on cattle abortion require a focus on previously understudied fertility after abortion in the first trimester of gestation, especially when abortion predisposes to opportunistic bacterial invasion of the uterus during the postpartum period, which may be attributed to immunosuppression. The investigation of hormonal, hematological, and oxidative profiles provides a general picture of the body’s conditions after abortion. OS and neutropenia are the most important findings in this study. OS may occur due to abortion itself or due to the invasion of opportunistic bacteria into the uterus after abortion, which eventually causes a reduction in fertility. Further studies are required to focus on the role of neutropenia in the pathogenesis of abortion to illustrate whether neutropenia acts as a contributor to the pathogenesis of abortion or if it is a later consequence of abortion, after other inflammatory changes in the blood. Furthermore, researching the mechanism causing this hematological shift, as well as any potential genetic susceptibility to this disorder, would be intriguing.

## List of abbreviations

CLSI, Clinical and Laboratory Standards Institute; NCCLS, National Committee for Clinical Laboratory Standards NRC, National Research Council; RBC, Red blood cell count; PCV, Packed cell volume; Hb, Hemoglobin; MCV, Mean corpuscular volume; MCH, Mean corpuscular hemoglobin; MCHC, Mean corpuscular hemoglobin concentration; WBC, White blood cell count; Ig, Immunoglobulin; P4, Progesterone; E2, Estradiol; OT, Oxytocin; PGF2α, Prostaglandin F2 alpha; MDA, Malondialdehyde; SOD, Superoxide dismutase; GPx, Glutathione peroxidase; TAC, Total antioxidant capacity; MDR, Multidrug resistant; *E. coli*, *Escherichia coli*.
